# Plasma levels of growth differentiation factor-15 are associated with myocardial injury in patients undergoing off-pump coronary artery bypass grafting

**DOI:** 10.1038/srep28221

**Published:** 2016-06-17

**Authors:** Zhize Yuan, Haiqing Li, Quan Qi, Wenhui Gong, Cheng Qian, Rong Dong, Yi Zang, Jia Li, Mi Zhou, Junfeng Cai, Zhe Wang, Anqing Chen, Xiaofeng Ye, Qiang Zhao

**Affiliations:** 1 Department of Cardiac Surgery, Ruijin Hospital, Shanghai Jiaotong University School of Medicine, Ruijin Er Road 197, Shanghai, 200025, P.R. China; 2 Department of Anesthesiology, Ruijin Hospital, Shanghai Jiaotong University School of Medicine, Ruijin Er Road 197, Shanghai, 200025, P.R. China; 3 State Key Laboratory of Drug Research, Shanghai Institute of Materia Medica, Chinese Academy of Sciences, Shanghai 201203, P.R. China; 4 East China Normal University, Institutes for Advanced Interdisciplinary Research, North Zhongshan Road Campus: 3663 N. Zhongshan Rd., Shanghai 200062, P.R. China

## Abstract

Growth differentiation factor-15 (GDF-15) has recently emerged as a risk predictor in patients with cardiovascular diseases. We therefore aimed to investigate the role of GDF-15 in the occurrence of cardiac injury during off-pump coronary artery bypass grafting (OPCAB). 55 consecutive patients with coronary artery diseases were recruited in this prospective, observational study. All patients were operated for OPCAB surgery. Serial blood samples were collected preoperatively, 12 hours and 36 hours after surgery. GDF-15, together with C-reactive protein, cardiac troponin I, creatine kinase MB and N-terminal pro B-type natriuretic peptide levels in plasma were measured at each time-point. GDF-15 levels increased significantly at 12 hours after surgery, attaining nearly 2.5 times the baseline levels (p < 0.001). Postoperative GDF-15 levels correlated positively with cTnI (p = 0.003) and EuroSCORE II (p = 0.013). According to the ROC curves, postoperative plasma GDF-15 was found to be the best biomarker to predict perioperative cardiac injury, compared with cTnI, CK-MB and EuroSCORE II. Circulating GDF-15 is a promising novel biomarker for identifying perioperative myocardial injury in patients undergoing OPCAB.

Myocardial injury and dysfunction are common and severe complications occurring during the post-operative period in patients undergoing cardiac surgery, and are associated with adverse outcomes such as longer length of hospital stay and higher mortality^1^,^2^,^3^,^4^. Moreover, clinical studies have shown that myocardial injury may be associated with higher risk of developing heart failure in the long term^5^,^6^,^7^. There are multiple proposed mechanisms thought to be responsible for post-operative cardiac injury including, but not limited to, calcium overload, oxygen free radical formation, neutrophil-mediated myocardial and endothelial injury, progressive decline in microvascular flow to the reperfused myocardium. The main causes are cardiac ischemia due to hypothermia, reduced blood flow, hypotension in operation procedure, atheroembolism, and inflammatory response, which may contribute to ischemia-reperfusion injuries^8^. These factors are interconnected in the pathophysiology of post-operative myocardial injury. Early detection of perioperative clinical biomarkers may aid us in the timely diagnosis and management of myocardial injury.

Various biological and hemodynamic markers have been measured to estimate the pre-and post-operative risk of developing complications^9^. In clinical practice, serum cardiac troponin I (cTnI) and creatine kinase MB (CK-MB) has traditionally been used for the assessment of myocardial injury after cardiac surgery^10^,^11^. A sharply rise in serum cTnI and CK-MB has been regarded as a sign of acute myocardial infarction (AMI)^12^. In spite of its widespread use, serum CK-MB and cTnI remains a flawed diagnostic potential for myocardial injury. Among other disadvantages, serum CK-MB level is a function not only of myocardial injury, but also production mainly by skeletal muscle cells. Because of this point, conditions that affect muscle mass (such as strenuous exercise, age, gender, race) may influence the level and the degree of increase in serum CK-MB^13^. In addition, a rise in serum cTnI occurs relatively late in the course of postoperative myocardial injury^14^, which makes early diagnosis and intervention difficult when relied upon cTnI as the main diagnostic method.

Growth-differentiation factor-15 (GDF-15) was found to be a new stress-responsive member of the transforming growth factor-β superfamily that was known as macrophage inhibitory cytokine-1^15^. Cardiomyocytes weakly express this cytokine under physiological conditions^16^. In response to inflammation as well as oxidative stress and ischemia reperfusion (I/R), expression levels of GDF-15 rise significantly^17^. This biomarker thus seems particularly relevant in the setting of cardiac surgery procedure, in which most of these mechanisms are involved. Several multicenter clinical trials showed that GDF-15 can be regarded as a reliable biomarker of cardiovascular disease and chronic heart failure and is of independent prognostic value in predicting coronary artery disease (CAD), acute coronary syndromes (ACS) and heart failure (HF)^9^,^18^,^19^,^20^,^21^.

Off-pump coronary artery bypass grafting (OPCAB) is part of the procedural armamentarium of a growing proportion of cardiac surgeons worldwide, especially in Asia. Studies comparing high-risk patient between OPCAB vs on-pump CABG have proved better outcome with OPCAB patients^22^,^23^. In a study with large population of patients undergoing cardiac surgery, it has been shown that the addition of pre-operative plasma level of GDF-15 to the EuroSCORE and other cardiovascular risk markers such as NT-proBNP or hsTNT dramatically improves the prognostic value for post-operative mortality and morbidity mortality^2^. However, no thoroughly analyzed data exist regarding the potential interest of role and impact of GDF-15 in the occurrence of myocardial injury during OPCAB. The aim of the present study was to evaluate plasma levels of GDF-15 and their diagnostic validity in patients with CAD undergoing OPCAB procedure, and to assess its relations to biochemical baseline variables and clinical cardiac dysfunction.

## Results

### Basic clinical characteristics of patients

The preoperative clinical and laboratory information of the 55 patients enrolled in the study are listed in Table 1. The Mean age was 66.5 ± 9.1 years and males constituted 80% of the total subjects. Factors, such as hypertension, diabetes mellitus, dyslipidemia and smoking were 72.7%, 40.0%, 34.5% and 38.2% respectively. CRP were 0.49(0.16–1.26) mg/L (range 0.10–7.86 mg/L), creatinines were 88.3 ± 28.7 mmol/L (range 55.0–250.0 mmol/L), cTnI were 0.01(0.01–1.78) ng/mL, and NT-proBNP levels were 488.7 ± 532.4 ng/mL (range 5.0–2036.0 ng/l). All patients received off-pump cardiac surgery. The majority of patients (n = 39) underwent at least three bypass grafts. Preoperative LVEF (%) was 63.3 ± 7.1 and the mean EuroSCORE II was 2.2. During their hospital stay, 5(3.6%) participants developed Perioperative myocardial infarction (PMI). All of the patients were still alive on 31st July 2015.

### Correlation between plasma pre-SURG GDF-15 concentrations and baseline characteristics

**Figure 1:**
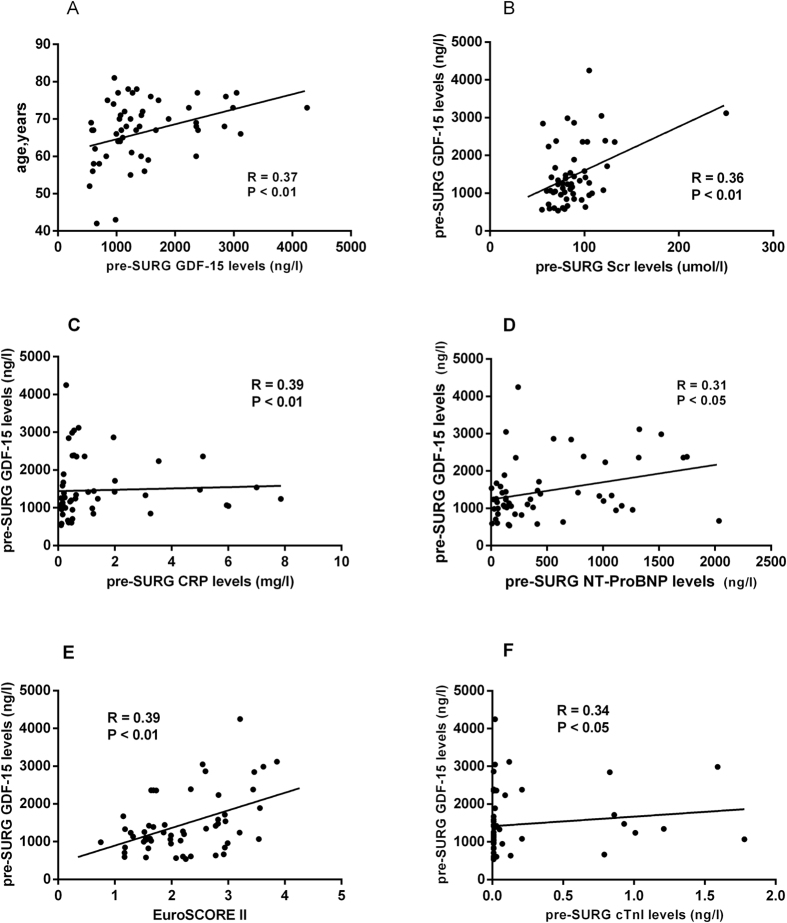
The correlation between pre-SURG circulating GDF-15 levels and baseline characteristics. Plasma GDF-15 was significant correlated with (**A**) age (r = 0.37; p = 0.005), (**B**) serum creatinine (r = 0.36; p = 0.007), (**C**) CRP (r = 0.39; p = 0.004), (**D**) NT-proBNP levels (r = 0.31, p = 0022), (**E**) EuroSCOREII (r = 0.39; p = 0.004), (**F**) cTnI (r = 0.34; p = 0.012).

**Figure 2:**
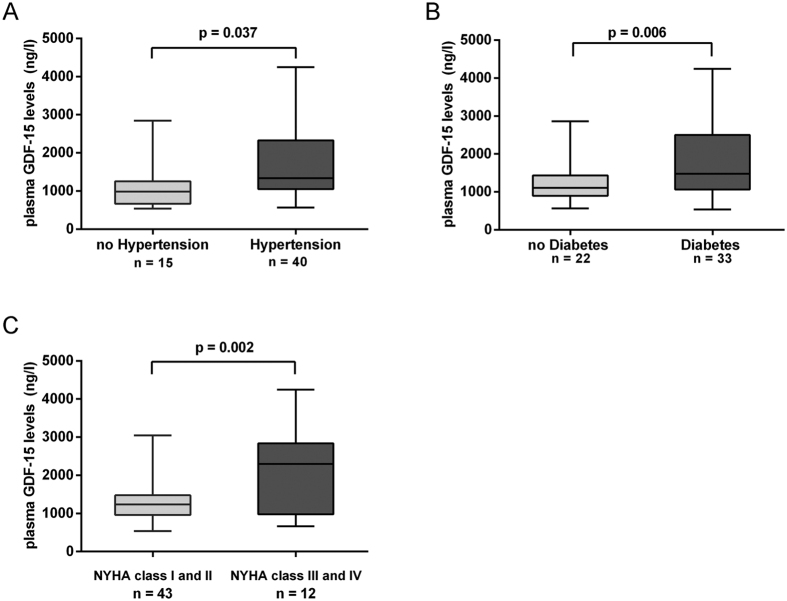
Association of pre-SURG circulating GDF-15 levels with clinical variables. GDF-15 levels were significantly related to the (**A**) hypertension (1600.8 ± 830.9 ng/l vs. 1097.6 ± 590.3 ng/l, p = 0.037) and (**B**) diabetes (1668 ± 269.1 ng/l vs. 1379 ± 123.5 ng/l, P = 0.006). Patients in NYHA functional class III or IV heart failure showed a significantly higher GDF-15 levels (2032.0 ± 1108.0 vs 1305.0 ± 619.0 ng/l, P = 0.002) as compared to those patients in NYHA functional class I and II (**C**) (Student’s test).

Mean GDF-15 levels were 1463.5 ± 800.1 ng/l (range 540.8–4249.7 ng/l). Associations between laboratory parameters and GDF-15 plasma concentrations were tested using Spearman’s correlation rank test. There was a significant correlation between pre-operative (pre-SURG) circulating levels of GDF-15 and the age (r = 0.37; P = 0.005, Fig. 1A), serum creatinine (r = 0.36; P = 0.007, Fig. 1B), CRP (r = 0.39; P = 0.004, Fig. 1C), NT-proBNP levels (r = 0.31, p = 0022, Fig. 1D), EuroSCORE II (r = 0.39; P = 0.004, Fig. 1E), and cTnI (r = 0.34; P = 0.012, Fig. 1F). In contrast, no significant correlation was observed between GDF-15 and LVEF (P = 0.23). Moreover, regarding categorical variables, GDF-15 levels were significantly related to the hypertension (1600.8 ± 830.9 ng/l vs. 1097.6 ± 590.3 ng/l, p = 0.037, Fig. 2A) and diabetes (1668 ± 269.1 ng/l vs. 1379 ± 123.5 ng/l, P = 0.006, Fig. 2B). However, plasma GDF-15 levels were insignificantly in patients with hyperlipemia (1340.4 ± 694.4 ng/l vs. 1528.5 ± 852.7 ng/l, P = 0.41). At enrollment, 12 of 55 patients were in NYHA functional class III or IV heart failure and showed a significantly higher GDF-15 levels (2032.0 ± 1108.0 vs 1305.0 ± 619.0 ng/l, P = 0.002) as compared to those patients in NYHA functional class I and II (Fig. 2C).

### Correlation between levels of post-SURG GDF-15 and extent of myocardial injury

**Figure 3:**
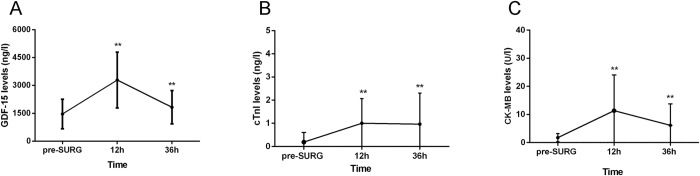
Time course of plasma GDF-15 levels (**A**), cTnI levels (**B**) and CK-MB levels (**C**) given as the mean ± SD. On average, GDF-15 levels, cTnI levels and CK-MB levels exhibited a 2.5- to 5-fold increase in plasma samples collected 12 h after OPCAB, respectively. **Significantly different (p < 0.01) between time-point and pre-SURG levels (One way repeated measures ANOVA).

**Figure 4:**
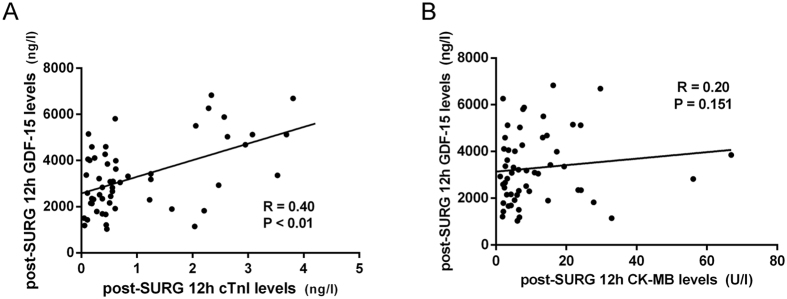
The correlation between post- SURG 12 h circulating GDF-15 levels and markers of cardiac injury. A significant correlation was observed between the post-SURG 12 h GDF-15 and cTnI (r = 0.40, P = 0.003) (**A**), However, an association statistic between the post-SURG 12 h GDF-15 and CK-MB shows no significant association (r = 0.20, P = 0.151) (**B**).

The postoperative clinical and laboratory information of the patients enrolled in the study are listed in Table 2. The circulating levels of GDF-15, as indicated by the fold change values, were highly elevated at 12 h after surgery in OPCAB patients. Mean post-operative 12 h, 36 h GDF-15 levels were 3293 ± 800.1 ng/l (range 1029.6–6832.9 ng/l) and 1828 ± 897.9 ng/l (range 600.9–4721.0 ng/l) respectively. The 12 h GDF-15 levels were 2.5 fold (P < 0.0001) higher than the pre-SURG control level (Fig. 3A). To further document an association between circulating concentrations of GDF-15 and myocardial injury, time course of simultaneous GDF-15, cTnI and CK-MB plasma levels were assessed (Fig. 3B,C). We correlated cTnI, CK-MB levels with GDF-15 levels. As illustrated in Fig. 4, a significant correlation was observed between the post-SURG 12 h GDF-15 and cTnI (r = 0.40, P = 0.003, Fig. 4A). However, an association statistic between the post-SURG 12 h GDF-15 and CK-MB shows no significant association (r = 0.20, P = 0.151, Fig. 4B). Thus, the release of the cardiac-enriched GDF-15 reflected the extent of myocardial injury as measured by cTnI release into circulation.

### Proposed predictive levels of circulating GDF-15 for PMI

**Figure 5:**
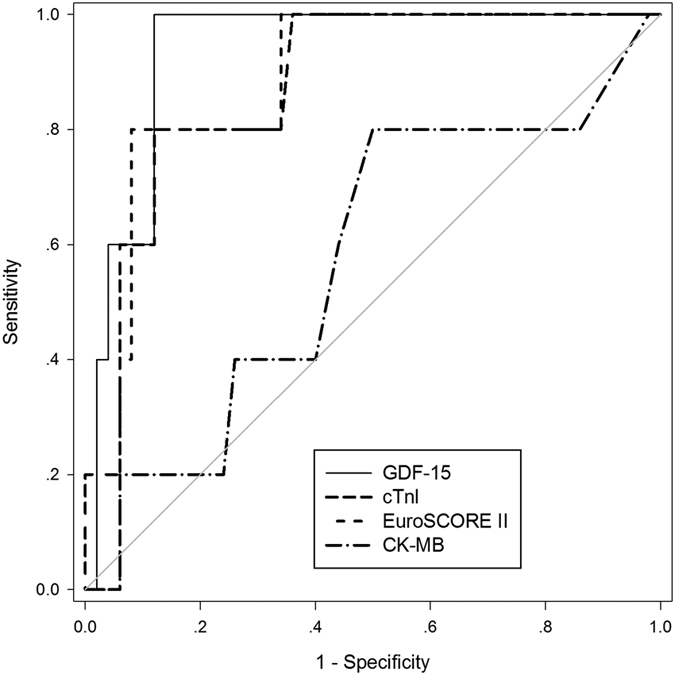
Receiver operating characteristic (ROC) curves comparing sensitivity and specificity of GDF-15, cTnI, CK-MB and EuroSCOREII for the identification of PMI at 12 h after OPCAB. The thin diagonal line is the Null Hypothesis: True area = 0.50. PMI was predicted by GDF-15 levels with an area under the curve (AUC) of 0.94 (95%CI 0.87–1.01, P = 0.001) and cTnI with an AUC of 0.88 (95% CI 0.75–1.02, P = 0.005), and EuroSCOREII with an AUC of 0.88 (95% CI 0.75–1.00, P = 0.006). As for CK-MB, for a cutoff value of 6.5U/l, the sensitivity was only 80.0% and the specificity 50.0%, with an area under the ROC curve of 0.576(0.28–0.87).

5 patients had PMI according to the diagnostic criteria of the Joint ESC/ACCF/AHA/WHF Task Force for the Third Universal Definition of Myocardial Infarction^24^: 5 had an elevation of cTnI > 10 × 99th percentile upper reference limit during the first 48 h, 3 had new pathological Q waves and 2 had new LBBB on ECG within 24 h, 2 had new regional wall motion abnormality within 12 h, and none had angiographic documented new graft or new native coronary artery occlusion. Univariate analysis revealed that cTnI, CK-MB, GDF-15, and EuroSCOREII were significantly associated with PMI. In a multivariate statistical model, GDF-15 was found to be significantly independently associated (p = 0.008). According to ROC curve analysis, PMI was predicted by GDF-15 levels with an area under the curve (AUC) of 0.94 (95%CI 0.87–1.01, P = 0.001) and cTnI with an AUC of 0.88 (95% CI 0.75–1.02, P = 0.005), and EuroSCOREII with an AUC of 0.88 (95% CI 0.75–1.00, P = 0.006) and is illustrated in Fig. 5. GDF-15 was a better postoperative plasma biomarker to predict PMI than cTnI or EuroSCOREII. As for CK-MB, for a cutoff value of 6.5U/l, the sensitivity was only 80.0% and the specificity 50.0%, with an area under the ROC curve of 0.576(0.28–0.87). GDF-15 was also substantially better than the CK-MB in predicting PMI. The optimal cutoff value of GDF-15 for the prediction of PMI was calculated to be 4638 ng/l with a sensitivity of 100.0% and a specificity of 88.0%.

As noted in Fig. 5, ROC analysis demonstrated the superior sensitivity and specificity of GDF-15 over CK-MB, cTnI and EuroSCOREII in the early detection of myocardial necrosis after OPCAB.

## Discussion

Cardiac surgery is associated with a systemic inflammatory response, which has implications for postoperative recovery and myocardial function^25^. Perioperative myocardial infarction (PMI) has been regarded as one of the severity complications for CABG and is associated with high morbidity and mortality for a long period of time^26^. Since no effective pharmacological therapy is available to treat PMI once occured, early preventive measures, including preoperative risk prediction and perioperative optimization may be a good way to reduce PMI^27^. Thus, PMI prediction thanks to a novel biomarker could provide valuable information in the perioperative management.

In experimental conditions, GDF-15 is widely expressed in cardiomyocytes, macrophages, adipocytes, vascular smooth muscle cells, and endothelial cells in pathological condition. GDF-15 expression is highly induced in cardiomyocytes after ischemia/reperfusion. GDF-15 inhibits the epidermal growth factor receptor (EGFR) activation and NF-*κ*B/JNK/caspase-3 pathway to provide its cardioprotective effect^28^.

Increased expression of GDF-15 was observed in the human heart within hours after myocardial infarction and remains elevated in the infarcted myocardium for several days. Cardiomyocytes in the infarct border zone have been identified as the main source of GDF-15^17^. In patients with diseases manifested by ineffective erythropoiesis, high GDF15 levels are present in the serum^29^. Catherine *et al*. reported a significant association between hemoglobin and GDF-15 levels during the post-operative period^30^.

In accordance with the clinical literature, some studies have shown that GDF-15 concentrations correlated strongly with age in adults^31^. Ho *et al*. reported that GDF-15 concentrations in healthy younger adults were lower than those in elderly adults^32^. It has been found that these changes of GDF-15 concentrations could reflect both cardiovascular inflammation and other pathophysiological processes^33^,^34^. The association between increased GDF-15 levels and diabetes has also been revealed. Several human studies dealing with GDF-15 levels in obesity and diabetes have shown that Serum GDF-15 levels were elevated in obese and type 2 diabetic patients and correlated with glucose and body mass index (BMI)^35^. Li, J. *et al*. proved that increased GDF-15 protects endothelial cells against glucose induced cellular injury via activating PI3 K/AKT/eNOS signaling pathway and attenuating NF-*κ*B/JNK activation^36^. In non-diabetic individuals, GDF-15 could predict future impaired glucose control and insulin resistance, and in population free of clinically-overt cardiovascular disease, GDF-15 levels are positively associated with age, diabetes, hypertension, creatinine and NTproBNP levels^32^. Indeed, several studies have also reported higher plasma GDF-15 levels in patients with cardiovascular pathologies such as coronary artery disease (CAD)^18^,^19^,^33^ or chronic heart failure^20^,^37^.

GDF-15 regulates signaling pathways for the essential of cardioprotection. GDF-15 activates Smad1 and reduces apoptotic cell death via upregulation of Bcl-xL and *β*-catenin. GDF-15 shows cardiac protective effect by activation of ALK type 1 receptors and phosphorylation of Smad2/3 and Smad1/5/8^38^. GDF-15 was a reliable biomarker of fatal events in patients with acute myocardial infarction. Furthermore, in patients with CAD, GDF-15 concentrations correlated with other biomarkers of inflammation. GDF-15 is associated with NT-proBNP and cTnT levels at presentation in those myocardial infarction patients^33^. Pre-operative GDF-15 plasma levels are also associated with post-operative AKI in CABG patients^39^. GDF-15 even improved risk stratification using the EuroSCORE before cardiac surgery^2^. Interestingly, Charles *et al*. clearly showed that preoperative GDF-15 levels increased a potential new predictive value to classic risk factors of postoperative atrial fibrillation in CABG and OPCAB^40^.

However, in the particular setting of PMI, very few studies are available regarding its predict value. Common factors of acute organ dysfunction after cardiac surgery have been described such as chronic obstructive pulmonary disease, hypertension, diabetes, peripheral vascular disease, congestive heart failure, cardiogenic shock, and on-pump surgery. Most of these predictive criteria were also found in our population except for on-pump surgery. The present study showed that pre-operative GDF-15 levels in patients undergoing OPCAB were positively associated with age, hypertension, diabetes, Scr, EuroSCOREII, NT-proBNP, hemoglobin, and plasma CRP levels. This is in agreement with previous researchs showing that GDF-15 was a novel independent biomarker in cardiovascular disease^21^,^34^. Apart from the preoperative clinical markers, we also found a considerable positive association between GDF-15 and cTnI during the post-operative period. In terms of the present study, it was interesting to note that there was no substantial positive correlation between GDF-15 and CK-MB. Persisting elevated GDF-15 levels closely related to myocardial injury, revealed by the plasma levels of cTnI. However, to our knowledge, this is the first study to report that elevated GDF-15 levels are strongly associated with biomarkers of myocardial injury after off-pump cardiac surgery. Moreover, despite its positive association with traditional risk factors of PMI in our study, the association between GDF-15 and PMI was independent of these risk factors.

During the cardiac surgery, multiple factors can lead to perioperative myocardial injury, including ischemia-reperfusion injury and cardiac dysfunction^41^. During the perioperative period, nonphysiological changes may be induced by changes in morphology and function of coronary plaque, which may trigger a mismatch between myocardial oxygen demand and supply^42^ contributing to myocardial ischemia and the cumulative release of biomarkers for myocardial injury. The correct clinical diagnosis of PMI is lagging, and very few studies concerning it associated with OPCAB. Our study demonstrated the superior sensitivity and specificity of GDF-15 over cTnI and EuroSCOREII in the early detection of myocardial necrosis after OPCAB. We believe that it is valuable to measure and identify GDF-15 as a more effective and sensitive biomarker than troponin to diagnose PMI, enabling more accurate therapeutic interventions and reducing postoperative morbidity and mortality.

## Conclusions

A perioperative measurement of plasma GDF-15 levels added a significant predictive value to classical risk factors of cardiac surgery. In patients with GDF-15 levels exceeding 4638 ng/l, further cardiac diagnosis and medical intervention should be considered because myocardial infarction or myocardial necrosis may be indicated. GDF-15 can be used as a novel biomarker for the detection of PMI of the OPCAB surgery in patients with coronary artery disease.

## Materials and Methods

### Study design

This study was designed as a prospective observational cohort study. The independent Medical Ethics Committee of the Ruijin Hospital, Shanghai Jiaotong University School of Medicine approved the study protocol according to the Helsinki declaration. Written informed consent was obtained from all patients before enrollment. The experiment methods were carried out in accordance with the approved guidelines and regulations.

### Patients population

A total of 55 consecutive patients between February to June 2015 at the Department of Cardiac Surgery, Ruijin Hospital, Shanghai Jiaotong University School of Medicine, Shanghai, China were included in this prospective study. Patients with a Left ventricular ejection fraction (LVEF) ≥40%, left ventricular end-diastolic diameter(LVED) ≤60 mm, without diffuse disease in target coronary artery are prone to be performed the revascularization with off pump. Patients with a porcelain or atherosclerotic aorta in whom there is increased risk for aortic trauma or cerebral embolization are also advocated revascularized with the use of OPCAB techniques. All patients operated on by 3 experienced surgeons (ZQ, CAQ, WZ) for OPCAB surgery procedure. The following criteria led to the exclusion of patients: refusal to provide consent, age <18 or >80 years, previous cardiac surgery and emergency surgery, primary and secondary myocardiopathy, combination with congenital heart disease, ACS reported within 30 days before the surgery, inflammatory disease or autoimmune disease, infectious or malignant tumor, transplant patients and patients treated with corticosteroids.

### Data collection

Clinical data of the enrolled patients collected from medical records was exhaustive, and the following variables were recorded: age, sex, usual cardiovascular risk factors, cardiovascular and pulmonary diseases, previous regular medication, and echocardiographic parameters. All of the patients underwent preoperative transthoracic echocardiography (TTE). The Echocardiographic data sets were assessed by investigators blinded to the laboratory results. LVEF was calculated using the Simpson method on the apical four-chamber and apical two-chamber views.

### Anesthesia and cardiac surgery procedure

Patients were pre-medicated with Alprazolam orally 12 h before anesthesia. Routine cardiac medications such as β-blockers, angiotensin converting enzyme (ACE) inhibitors, angiotensin receptor blockers (ARB), calcium channel blockers (CCB), and lipid lowering drugs were continued until the morning of the surgery, except for aspirin and clopidogrel, which were stopped at least 1 day earlier. After a radial artery hemodynamic monitoring system was set up in the operating room. Anesthesia was induced with intravenous midazolam (0.03 mg/kg), sufentanil (0.2 to 0.5 mg/kg/h), and propofol (1.5 to 2.5 mg/kg). After verifying that manual ventilation was satisfactory, cisatracurium (0.06 mg/kg/h) was injected. Patients were orally intubated and ventilated with FiO_2_: 40%. Anesthesia was maintained with sufentanil and cisatracurium as required and inhaled desflurane.

Following general anesthesia and median sternotomy, the left internal mammary artery (LIMA) and saphenous vein graft were harvested simultaneously in all of the patients. Off-pump surgery procedure was performed on the beating heart utilizing a stabilizer (Octopus Medtronic, MN, USA) and an intracoronary shunt (Guidant Axius Coronary Shunt, Guidant Corporation, Santa Clara, CA, USA). Patients were heparinized intravenously to achieve an activated clotting time >300 s. A side-biting aorta clamp was usually used when proximal anastomoses were performed. Flow measurements are carried out with transit time flow probes (Medtronic, Inc, USA). After closure of the sternum, patients were transferred safety to the postoperative intensive care unit (ICU).

### Blood sampling and Biochemical analyses

Blood samples of the enrolled patients were collected and analyzed for routine blood testing on the second day morning of hospital admission (Pre-SURG) were used as baseline reference values. The following measurements were made: CK-MB, cTnI, N-terminal pro-B-type natriuretic peptide (NT-proBNP), C-reactive protein (CRP), hemoglobin, blood glucose, total plasma cholesterol, triglyceride, low-density lipoprotein cholesterol and creatinine. Plasma levels of cTnI were determined by electrochemiluminescence-based methods with a Beckman ACCESS2 Analyzer. The upper limit for the normal reference range was 0.04 ng/l. Biochemical measurements were performed using standard laboratory techniques. All biochemical analyses were performed by investigators blinded to the clinical data of the patients.

GDF-15 levels in plasma were measured on the Pre-SURG time point, 12 h and 36 h after arrival at the ICU (Post-SURG). From the patients, 3 mL blood was collected from the median cubital vein of the patients into a tube containing EDTA anticoagulant. After incubation at room temperature for 2 h, Blood samples were centrifuged and the plasma was immediately frozen in liquid nitrogen and stored at −80 °C until measurement. Plasma GDF-15 concentrations were measured in duplicate by quantitative sandwich enzyme immunoassay (Quantikine, R&D Systems, USA). The color intensity, relative to GDF-15 concentration, was measured at 450 nm with a spectrophotometer (BioTek, Winooski, VT, United States).

### Statistical analyses

Data were analyzed using standard statistical software SPSS version 19.0 (SPSS Inc, Chicago, Illinois, USA) and GraphPad Prism 6.0 software (GraphPad Software Inc, California, USA). Continuous variables are presented as means ± standard deviations (SD) unless otherwise indicated; categorical variables as numbers (percentages). For continuous data, normality was checked by the Kolmogorov–Smirnov test. Non parametric variables were reported as medians with interquartile ranges (IQR). Differences between unpaired groups were analyzed using a Mann-Whitney U test for continuous variables and a χ2 test (or Fisher exact test, if numbers were small) for dichotomous variables. A double-sided p-value < 0.05 was considered statistically significant for all tests. Associations between laboratory parameters and GDF-15 were tested using Spearman’s correlation rank test. To examine the discrimination of myocardial infarction, we examined the area under the receiver-operating characteristic (ROC) Curve (plot of sensitivity versus 1−specificity for all possible cut-off values for classifying predictions) for post-SURG 12 h GDF-15, cTnI, CK-MB and EuroSCOREII with the best sensitivity and specificity according to the Youden index^43^. The cut-off value is given in the Results section. Areas under the ROC curves were compared according to the methods described by DeLong *et al*.^44^ for paired data.

## Additional Information

**How to cite this article**: Yuan, Z. *et al*. Plasma levels of growth differentiation factor-15 are associated with myocardial injury in patients undergoing off-pump coronary artery bypass grafting. *Sci. Rep.* **6**, 28221; doi: 10.1038/srep28221 (2016).

## Figures and Tables

**Table 1:** Demographic and Perioperative Characteristics of Enrolled Patients.

Baseline parameter	n(%), median (interquartile range), mean ± SD	P value
Clinical data		
Age, years	66.5 ± 9.1	P = 0.005
Male Gender	44 (80.0)	P = 0.073
Body mass index, kg/m^2^	23.1 ± 2.4	P = 0.025
Systemic hypertension	40(72.7)	P = 0.037
Diabetes mellitus	22(40)	P = 0.006
hyperlipidemia	19(34.5)	P = 0.410
Smoking	21(38.2)	P = 0.940
CCS	2(2–3)	P = 0.591
NYHA	2(2–3)	P = 0.458
Prior myocardial infarction	10(18.2)	P = 0.134
Preoperative LVEF (%)	63.3 ± 7.1	P = 0.229
EuroSCOREII	2.2 ± 0.8	P = 0.004
Previous treatments		
Aspirin	53(96.4)	
Beta blockers	43(78.2)	
Statins	40(72.7)	
ACE inhibitors	39(70.9)	
Preoperative Biological data		
Serum creatinine, mmol/L	88.3 ± 28.7	P = 0.007
Haemoglobin	130.0 ± 14.3	P = 0.005
NT-proBNP, ng/L	488.7 ± 532.4	P = 0.022
Troponin I, ng/ml	0.01(0.01–0.07)	P = 0.012
Creatine kinase MB, U/l	1.7 ± 1.4	P = 0.372
CRP, mg/L	0.49(0.16–1.26)	P = 0.040

BMI, body mass index; OPCAB, off-pump coronary artery bypass; CCS, Canadian classification score for angina grade; NYHA, New York Heart Association grade for heart failure; ACE, angiotensinconverting enzyme; LVEF, left ventricular ejection fraction; EuroSCOREII, European System for Cardiac Operative Risk Evaluation II; ICU, intensive care unit; CAD, Coronary artery disease; CRP, C reactive protein.

**Table 2:** The postoperative clinical and laboratory information of the patient.

Postoperative parameter	n(%), median (interquartile range), mean ± SD	P value
Distal coronary anastomose (no./patient)	3 (2–4)	P = 0.302
Haemoglobin	111.8 ± 16.6	P = 0.175
Troponin I, ng/ml	1.0 ± 1.1	P = 0.001
Creatine kinase MB, U/l	11.3 ± 12.7	P = 0.151
Postoperative LVEF (%)	63.3 ± 7.1	P = 0.146
In-hospital stay after surgery (days)	8.1 ± 2.9	P = 0.408
ICU stay (days)	2.2 ± 0.7	P = 0.018
Postoperative atrial fibrillation	7(12.7)	P = 0.090

## References

[b1] Breisblatt W. M. . Acute myocardial dysfunction and recovery: a common occurrence after coronary bypass surgery. J. Am. Coll. Cardiol. 15, 1261–1269 (1990). 2109763 10.1016/s0735-1097(10)80011-7

[b2] Heringlake M. . Growth differentiation factor 15: a novel risk marker adjunct to the EuroSCORE for risk stratification in cardiac surgery patients. J. Am. Coll. Cardiol. 61, 672–681 (2013). 23391200 10.1016/j.jacc.2012.09.059

[b3] Selvanayagam J. B. . Effects of Off-Pump Versus On-Pump Coronary Surgery on Reversible and Irreversible Myocardial Injury A Randomized Trial Using Cardiovascular Magnetic Resonance Imaging and Biochemical Markers. Circulation 109, 345–350 (2004). 14732755 10.1161/01.CIR.0000109489.71945.BD

[b4] Zhang X. . Analysis of the Bypass Angioplasty Revascularization Investigation Trial Using a Multistate Model of Clinical Outcomes. The American journal of cardiology 115, 1073–1079 (2015). 25724784 10.1016/j.amjcard.2015.01.543 PMC4380580

[b5] Perna E. R. . Ongoing myocardial injury in stable severe heart failure value of cardiac troponin T monitoring for high-risk patient identification. Circulation 110, 2376–2382 (2004). 15477403 10.1161/01.CIR.0000145158.33801.F3

[b6] Saunders J. T. . Cardiac troponin T measured by a highly sensitive assay predicts coronary heart disease, heart failure, and mortality in the Atherosclerosis Risk in Communities Study. Circulation 123, 1367–1376 (2011). 21422391 10.1161/CIRCULATIONAHA.110.005264 PMC3072024

[b7] Missov E., Calzolari C. & Pau B. Circulating cardiac troponin I in severe congestive heart failure. Circulation 96, 2953–2958 (1997). 9386162 10.1161/01.cir.96.9.2953

[b8] Yellon D. M. & Hausenloy D. J. Myocardial reperfusion injury. N. Engl. J. Med. 357, 1121–1135 (2007). 17855673 10.1056/NEJMra071667

[b9] Preeshagul I. . Potential biomarkers for predicting outcomes in CABG cardiothoracic surgeries. J. Cardiothorac. Surg. 8, 176 (2013). 23866777 10.1186/1749-8090-8-176 PMC3726492

[b10] Croal B. L. . Relationship between postoperative cardiac troponin I levels and outcome of cardiac surgery. Circulation 114, 1468–1475 (2006). 17000912 10.1161/CIRCULATIONAHA.105.602370

[b11] Brener S. J., Lytle B. W., Schneider J. P., Ellis S. G. & Topol E. J. Association between CK-MB elevation after percutaneous or surgical revascularization and three-year mortality. J. Am. Coll. Cardiol. 40, 1961–1967 (2002). 12475456 10.1016/s0735-1097(02)02538-x

[b12] Costa M. A. . Incidence, predictors, and significance of abnormal cardiac enzyme rise in patients treated with bypass surgery in the arterial revascularization therapies study (ARTS). Circulation 104, 2689–2693 (2001). 11723020 10.1161/hc4701.099789

[b13] Brancaccio P., Maffulli N. & Limongelli F. M. Creatine kinase monitoring in sport medicine. Br. Med. Bull. 81-82, 209–230 (2007). 17569697 10.1093/bmb/ldm014

[b14] Wu A., Feng Y. J., Contois J. H. & Pervaiz S. Comparison of myoglobin, creatine kinase-MB, and cardiac troponin I for diagnosis of acute myocardial infarction. Ann. Clin. Lab. Sci. 26, 291–300 (1996). 8800429

[b15] Bootcov M. R. . MIC-1, a novel macrophage inhibitory cytokine, is a divergent member of the TGF-β superfamily. Proceedings of the National Academy of Sciences 94, 11514–11519 (1997). 10.1073/pnas.94.21.11514 PMC23523 9326641

[b16] Shi Y. & Massagué J. Mechanisms of TGF-β signaling from cell membrane to the nucleus. Cell 113, 685–700 (2003). 12809600 10.1016/s0092-8674(03)00432-x

[b17] Kempf T. . The transforming growth factor-β superfamily member growth-differentiation factor-15 protects the heart from ischemia/reperfusion injury. Circ. Res. 98, 351–360 (2006). 16397141 10.1161/01.RES.0000202805.73038.48

[b18] Wollert K. C. . Prognostic value of growth-differentiation factor-15 in patients with non–ST-elevation acute coronary syndrome. Circulation 115, 962–971 (2007). 17283261 10.1161/CIRCULATIONAHA.106.650846

[b19] Khan S. Q. . Growth differentiation factor-15 as a prognostic marker in patients with acute myocardial infarction. Eur. Heart J. 30, 1057–1065 (2009). 19168526 10.1093/eurheartj/ehn600

[b20] Kempf T. . Prognostic utility of growth differentiation factor-15 in patients with chronic heart failure. J. Am. Coll. Cardiol. 50, 1054–1060 (2007). 17825714 10.1016/j.jacc.2007.04.091

[b21] Lind L. . Growth-differentiation factor-15 is an independent marker of cardiovascular dysfunction and disease in the elderly: results from the Prospective Investigation of the Vasculature in Uppsala Seniors (PIVUS) Study. Eur. Heart J., ehp261 (2009). 10.1093/eurheartj/ehp261 19561023

[b22] Lamy A. . Off-pump or on-pump coronary-artery bypass grafting at 30 days. N. Engl. J. Med. 366, 1489–1497 (2012). 22449296 10.1056/NEJMoa1200388

[b23] Lamy A. . Effects of off-pump and on-pump coronary-artery bypass grafting at 1 year. N. Engl. J. Med. 368, 1179–1188 (2013). 23477676 10.1056/NEJMoa1301228

[b24] Thygesen K. . Third universal definition of myocardial infarction. J. Am. Coll. Cardiol. 60, 1581–1598 (2012). 22958960 10.1016/j.jacc.2012.08.001

[b25] Laffey J. G. The systemic inflammatory response to cardiac surgery: implications for the anesthesiologist. Anesthesiology 97, 215–252 (2002). 12131125 10.1097/00000542-200207000-00030

[b26] Namay D. L. . Effect of perioperative myocardial infarction on late survival in patients undergoing coronary artery bypass surgery. Circulation 65, 1066–1071 (1982). 6122512 10.1161/01.cir.65.6.1066

[b27] Force T. . Perioperative myocardial infarction after coronary artery bypass surgery. Clinical significance and approach to risk stratification. Circulation 82, 903–912 (1990). 2394010 10.1161/01.cir.82.3.903

[b28] Ho F. M. . High glucose-induced apoptosis in human vascular endothelial cells is mediated through NF-kappaB and c-Jun NH2-terminal kinase pathway and prevented by PI3K/Akt/eNOS pathway. Cell Signal 18, 391–399 (2006). 15970429 10.1016/j.cellsig.2005.05.009

[b29] Tanno T. . High levels of GDF15 in thalassemia suppress expression of the iron regulatory protein hepcidin. Nat Med 13, 1096–1101 (2007). 17721544 10.1038/nm1629

[b30] Kahli A. . Growth differentiation factor-15 (GDF-15) levels are associated with cardiac and renal injury in patients undergoing coronary artery bypass grafting with cardiopulmonary bypass. PloS one 9, e105759 (2014). 25171167 10.1371/journal.pone.0105759 PMC4149498

[b31] Eggers K. M. . Growth-differentiation factor-15 for long-term risk prediction in patients stabilized after an episode of non–ST-segment–elevation acute coronary syndrome. Circ. Cardiovasc. Genet. 3, 88–96 (2010). 20160200 10.1161/CIRCGENETICS.109.877456

[b32] Ho J. E. . Clinical and genetic correlates of growth differentiation factor 15 in the community. Clin. Chem. 58, 1582–1591 (2012). 22997280 10.1373/clinchem.2012.190322 PMC4150608

[b33] Kempf T. . Growth-differentiation factor-15 improves risk stratification in ST-segment elevation myocardial infarction. Eur. Heart J. 28, 2858–2865 (2007). 17977844 10.1093/eurheartj/ehm465

[b34] Eggers K. M., Kempf T., Wallentin L., Wollert K. C. & Lind L. Change in growth differentiation factor 15 concentrations over time independently predicts mortality in community-dwelling elderly individuals. Clin. Chem. 59, 1091–1098 (2013). 23529704 10.1373/clinchem.2012.201210

[b35] Ding Q. . Identification of macrophage inhibitory cytokine-1 in adipose tissue and its secretion as an adipokine by human adipocytes. Endocrinology 150, 1688–1696 (2009). 19074584 10.1210/en.2008-0952

[b36] Li J. . Adaptive induction of growth differentiation factor 15 attenuates endothelial cell apoptosis in response to high glucose stimulus. PloS one 8, e65549 (2013). 23799024 10.1371/journal.pone.0065549 PMC3683015

[b37] Kempf T. & Wollert K. C. Growth-differentiation factor-15 in heart failure. Heart Fail. Clin. 5, 537–547 (2009). 19631178 10.1016/j.hfc.2009.04.006

[b38] Ago T. & Sadoshima J. GDF15, a cardioprotective TGF-beta superfamily protein. Circ Res 98, 294–297 (2006). 16484622 10.1161/01.RES.0000207919.83894.9d

[b39] Guenancia C. . Pre-operative growth differentiation factor 15 as a novel biomarker of acute kidney injury after cardiac bypass surgery. Int J Cardiol 197, 66–71 (2015). 26113476 10.1016/j.ijcard.2015.06.012

[b40] Bouchot O. . Low Circulating Levels of Growth Differentiation Factor-15 Before Coronary Artery Bypass Surgery May Predict Postoperative Atrial Fibrillation. J Cardiothorac Vasc Anesth 29, 1131–1139 (2015). 25990268 10.1053/j.jvca.2015.01.023

[b41] Birdi I., Angelini G. D. & Bryan A. J. Biochemical markers of myocardial injury during cardiac operations. The Annals of thoracic surgery 63, 879–884 (1997). 9066431 10.1016/s0003-4975(96)01275-1

[b42] Priebe H.-J. Perioperative myocardial infarction—aetiology and prevention. Br. J. Anaesth. 95, 3–19 (2005). 15665072 10.1093/bja/aei063

[b43] Youden W. J. Index for rating diagnostic tests. Cancer 3, 32–35 (1950). 15405679 10.1002/1097-0142(1950)3:1<32::aid-cncr2820030106>3.0.co;2-3

[b44] DeLong E. R., DeLong D. M. & Clarke-Pearson D. L. Comparing the areas under two or more correlated receiver operating characteristic curves: a nonparametric approach. Biometrics 44, 837–845 (1988). 3203132

